# In vivo corneal confocal microscopy and optical coherence tomography on eyes of participants with type 2 diabetes mellitus and obese participants without diabetes

**DOI:** 10.1007/s00417-021-05251-8

**Published:** 2021-07-20

**Authors:** Noémi Tóth, David M. Silver, Szabolcs Balla, Miklós Káplár, Adrienne Csutak

**Affiliations:** 1 Department of Ophthalmology, University of Pécs, Medical School, Akác u. 1, Pécs, 7623 Hungary; 2 Department of Ophthalmology, Faculty of Medicine, University of Debrecen, Debrecen, 4032 Hungary; 3 Doctoral School of Clinical Medicine, University of Debrecen, Debrecen, 4032 Hungary; 4 Johns Hopkins University, Bethesda, MA 20816 USA; 5 Division of Metabolic Diseases, Department of Internal Medicine, Faculty of Medicine, University of Debrecen, Debrecen, 4032 Hungary

**Keywords:** Type 2 diabetes mellitus, Obesity, In vivo confocal corneal microscopy, OCT

## Abstract

**Abstract:**

**Purposes:**

To examine corneal nerve and retinal nerve characteristics of participants with type 2 diabetes mellitus (T2DM) compared with obese participants without diabetes to discover potential nerve vulnerabilities.

**Methods:**

All participants underwent a complete medical examination including a physical examination and blood sample tests. The ophthalmologic examination included best-corrected visual acuity, intraocular pressure, Schirmer test, tear film breakup time, slit-lamp examination, dilated fundus photography, in vivo corneal confocal microscopy (IVCCM), and optical coherence tomography (OCT).

**Results:**

The study cohort consisted of 83 eyes of 83 individuals: a group of 44 participants with T2DM, and a control group of 39 obese participants with no history of diabetes. Comparing measurements on the two groups, participants with T2DM had lower values with statistical significance for retinal nerve fiber layer (RNFL) nasal superior thickness (*p* = 0.010) and three corneal nerve (CN) parameters: fiber length (*p* = 0.025), total branch density (*p* = 0.013), and fiber area (*p* = 0.009). There was a borderline significant difference in CN fiber width (*p* = 0.051) and RNFL nasal inferior thickness (*p* = 0.056). No other significant differences were observed in the IVCCM and OCT parameters. No statistically significant correlation was found between CN and RNFL parameters.

**Conclusions:**

Progression from a pre-diabetic obese state to a T2DM condition might entail a loss or diminishment of certain corneal nerve fibers or retinal nerve fibers, but not necessarily a loss of both corneal and retinal nerve fibers simultaneously. Using IVCCM and OCT together enables monitoring of both corneal and retinal health of the eye.

**Supplementary Information:**

The online version contains supplementary material available at 10.1007/s00417-021-05251-8.

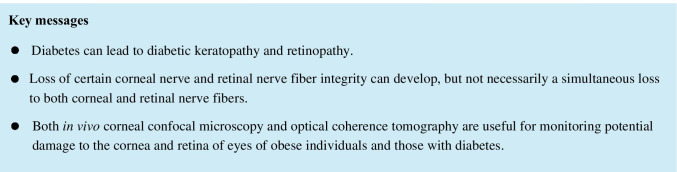



## Introduction

Chronically existing hyperglycemia and consequential vascular complications are the main diabetic side effects causing mortality and disability [1]. Diabetes mellitus regularly results in corneal edema, erosions and changes of the tear film, and worsening corneal epithelial wound healing, despite the fact that it remains undetected clinically in almost 70% of the cases [2, 3]. Neuropathy can be diagnosed at an early stage with in vivo corneal confocal microscopy (IVCCM), which is a non-invasive technique that allows the clinician to examine the layers of the cornea accurately [4–6]. In addition, optical coherence tomography (OCT) plays a key role in identifying and quantifying the grade of maculopathy in diabetes mellitus [7]. Attention has also been focused on retinal nerve fiber layer (RNFL) and choroidal layer thickness measurements [8–10].

In a previous work, RNFL thickness has been associated with a range of health and lifestyle parameters [11]. Reduced corneal nerve fiber size has been observed in people with diabetes [12–14]. IVCCM on individuals with type 2 diabetes mellitus (T2DM) has demonstrated increased Langerhans cells and decreased sub-basal nerve plexus that may be related to lower basal endothelial cell density and may be responsible for corneal epithelium healing delay [15].

The aim of this study was to examine the differences between corneal and retinal nerve fiber characteristics of participants with T2DM compared with obese controls without diabetes using IVCCM and OCT to discover potential nerve vulnerabilities accompanying diabetes and obesity.

## Participants and methods

### Study participants

The study cohort consisted of 83 eyes of 83 individuals: a group of 44 participants with T2DM, 26 males and 18 females; and a control group of 39 obese participants with no history of diabetes, 16 males and 23 females. The eyes of all individuals were examined in the Ophthalmology Department, Faculty of Medicine, University of Debrecen. When both eyes were suitable for the study, one was chosen randomly. Inclusion and exclusion criteria are listed in Table 1.

**Table 1:** Inclusion and exclusion criteria for participation in the study

Inclusion criteria	Exclusion criteria
Written informed consent form	Previous intraocular surgery
Age > 18 years	Glaucoma
BCVA = 20/20 ft. (Snellen)	Contact lens wear
T2DM group: diabetes diagnosis	Diabetic maculopathy
Obese group: BMI > 30.0 kg/m^2^	Proliferative diabetic retinopathy

*BCVA* best-corrected visual acuity, *T2DM* type 2 diabetes mellitus, *BMI* body mass index

Ethical permission was granted by the University of Debrecen Ethics Committee (No. OGYÉI/2829/2017) and all participants provided written informed consent in accordance with the Helsinki Declaration.

### Clinical and ophthalmological investigations

All participants underwent a complete medical examination including a physical examination and blood sample tests. Tabulated values for the 83 participants included age, body mass index (BMI), glycated hemoglobin (HbA1c), glucose, triglyceride, cholesterol, high-density lipoprotein (HDL), and low-density lipoprotein (LDL). The ophthalmologic examination included best-corrected visual acuity (BCVA), intraocular pressure (IOP), Schirmer test, tear film breakup time (BUT), slit-lamp examination, dilated fundus photography, IVCCM, and OCT.

### In vivo corneal confocal microscopy

Heidelberg Retina Tomograph III Rostock Cornea Module (HRT III RCM, Heidelberg Engineering GmbH, Heidelberg, Berlin, Germany) was used during the IVCCM analysis. Tetracaine hydrochloride 0.5% was administered as local anesthesia and the participant was focusing on a distant target before the central cornea was scanned. Section and volume scans were recorded from the basal epithelium anterior to Bowman’s layer, sub-basal nerve plexus, posterior stroma anterior to Descemet’s membrane, and endothelial cells. These observations did not include the morphology or density of Langerhans cells. Three representative corneal confocal microscopy (CCM) images were chosen for analysis, (a) basal epithelium (epithelial cells), (b) average of anterior and posterior stromal keratocytes (stromal keratocytes), and (c) endothelial cell layers (endothelial cells), all measured in number/mm^2^. A region of interest was defined as containing at least 50 cells, which were chosen from each cell layer. At least 5, on average 8, good quality [16] pictures from the central area were used to evaluate the sub-basal nerve plexus, not overlapping more than 20%.

The cells were identified and marked manually and the cell densities were automatically calculated by the instrument-based software (Heidelberg Eye Explorer software, Heidelberg Engineering GmbH, Heidelberg, Berlin, Germany). ACCMetrics software (version 2.0; University of Manchester, Manchester, UK) [17] was used to evaluate the morphology of the sub-basal nerve plexus. The execution and analysis of the examinations were performed by one masked examiner.

The following corneal nerve (CN) parameters were quantified: CN fiber density, the number of nerve fibers per unit area; CN branch density, the number of primary branch fibers per unit area; CN fiber length, the total length of nerves per unit area; CN total branch density, the total number of branch points per unit area; CN fiber area, the total nerve fiber area per unit area; CN fiber width, the average nerve fiber width per unit area; and CN fractal dimension.

### Optical coherence tomography

Retina scans were acquired with the SPECTRALIS OCT system (Heidelberg Engineering, Heidelberg, Germany). Volume scans (49 line, 30°, HS, ART 16) were performed for multi-segment volume measurements for foveal volume at the central 6 mm area. Line scans (30°, HR, ART 50) were used with enhanced depth imaging (EDI) for central retinal thickness, choroidal layer thickness, and built-in RNFL thickness measurements using the manual correction option for ideal centering on the optic nerve head. All examinations were performed and evaluated by one examiner. The built-in RNFL parameters were global thickness, temporal, temporal inferior, temporal superior, nasal, nasal inferior, and nasal superior.

### Statistical analysis

A total of 1909 ophthalmic measurements (23 measurement categories on each of 83 individuals) were considered in this work. Of these, 70 measurements were missing (measurement was not made) and 11 measurements were outliers that did not satisfy the Grubbs criteria [18] for inclusion, leaving 1828 measurements for further analysis. There were 664 non-ophthalmic measurements (8 categories on each of 83 participants) of which 8 measurements were missing and 7 measurements were outliers, giving 649 reliable measurements. In addition, age and gender were recorded. Normal distribution of samples within each measurement category was tested with analysis of residuals from a normal plot [19]. Equivalence of measurements within a given measurement category between T2DM and obese groups were tested with *t* tests, Mann-Whitney U tests, and one-way analysis of variance [20]; since these three analyses gave equivalent results, *t* tests were chosen to display herein. For correlation analyses, each pair of measurement categories was reduced to the minimum pairwise number of measurements in the pair. The Pearson product-moment correlation coefficient was used with the *t* test for statistical significance. Statistical significance was defined as *p* < 0.05.

## Results

### Demographic and clinical data

Age and BMI distributions among the two study groups are shown in Table 2. Participants in the obese group had highly significant (*p* < 0.001) increased BMI compared to participants with T2DM. Nevertheless, two-thirds of the participants with T2DM had BMI greater than 30 kg/m^2^, which would qualify them as obese.

**Table 2:** Age and body mass index of participants with type 2 diabetes mellitus (T2DM) and the obese control group without diabetes

	Age (years)			Body mass index (kg/m^2^)		
	T2DM	Obese	*p* value	T2DM	Obese	*p* value
Mean	50	53		33.2	38.6	
Std. Dev.	7	10		5.2	6.10	
Maximum	63	67	0.221	47.3	54.50	**< 0.001**
Median	51	52		32.4	36.5	
Minimum	33	32		24.6	31.0	
Number	44	39		43	39	

The *p* values were calculated using *t* tests

Bold values denote statistical significance at the *p* < 0.05 level

**Fig. 1:**
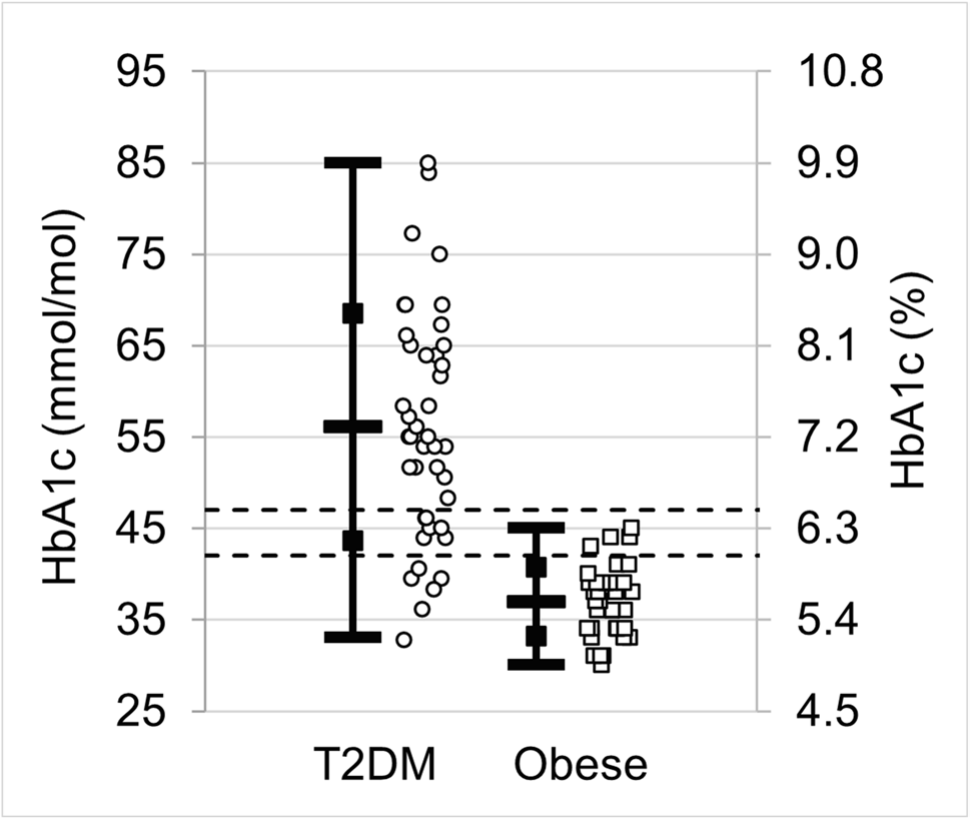
Measurements of HbA1c (left scale: mmol/mol, right scale: %) for the participants with type 2 diabetes mellitus (T2DM) and the obese control group without diabetes. The open bars on vertical lines indicate the mean value, open squares indicate ± standard deviation, and solid bars indicate minimum and maximum values. The raw data are shown scattered horizontally for clarity. Dashed lines are drawn at 42 and 47 mmol/mol (6.0 and 6.5%). T2DM, *N* = 42; obese, *N* = 39

**Fig. 2:**
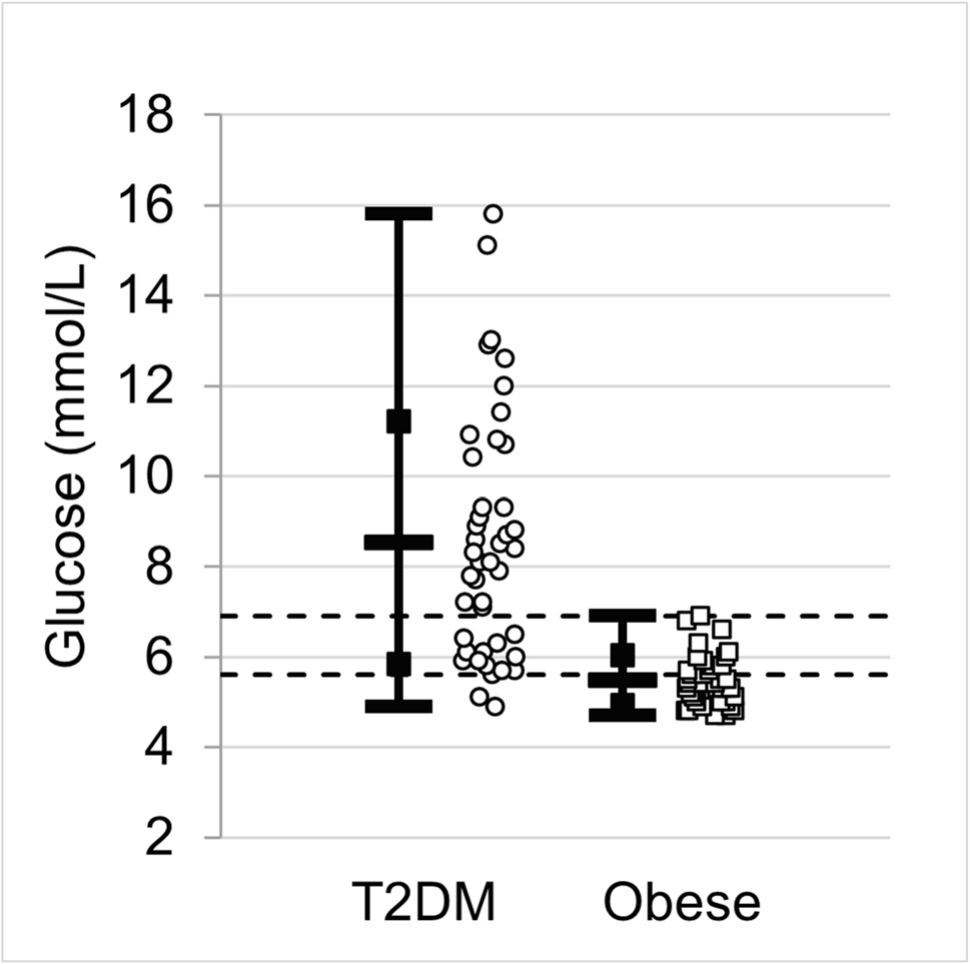
Measurements of glucose (mmol/L) for the participants with type 2 diabetes mellitus (T2DM) and the obese control group without diabetes. The open bars on vertical lines indicate the mean value, open squares indicate ± standard deviation, and solid bars indicate minimum and maximum values. The raw data are shown scattered horizontally for clarity. Dashed lines are drawn at 5.6 and 6.9 mmol/L. T2DM, *N* = 43; obese, *N* = 39

The results of blood sample measurements of HbA1c and glucose are displayed in Table 3 and the distribution of measurements is shown in Figs. 1 and 2, illustrating the difference between people with diabetes and those who are obese without diabetes. Eighteen of the diabetic patients were on insulin therapy but nevertheless had elevated HbA1c values.

**Table 3:** Blood sample measurements of participants with type 2 diabetes mellitus (T2DM) and the obese control group without diabetes

	HbA1c					Glucose		
	(mmol/mol)		(%)			(mmol/L)		
	T2DM	Obese	T2DM	Obese	*p* value	T2DM	Obese	*p* value
Mean	56	37	7.3	5.5		8.5	5.5	
Std. Dev.	12	4	1.1	0.3		2.7	0.6	
Maximum	85	45	9.9	6.3	**< 0.001**	15.8	6.9	**< 0.001**
Median	55	37	7.2	5.5		8.1	5.4	
Minimum	33	30	5.2	4.9		4.9	4.7	
Number	42	39	42	39		43	39	

The *p* values were calculated using *t* tests

Bold values denote statistical significance at the *p* < 0.05 level

HbA1c and glucose levels were higher for people in the T2DM group with the difference having high statistical significance (*p* < 0.001). Comparing the HbA1c measurements to standard reference ranges, 17% of people with T2DM and 90% of the people in the obese group had HbA1c below 42 mmol/mol; 12% with T2DM and 10% obese had HbA1c in the range 42–47 mmol/mol; and 71% with T2DM and 0% obese had HbA1c above 47 mmol/mol. Comparing glucose measurements to reference ranges, 7% of people with T2DM and 62% of obese controls had glucose below 5.6 mmol/L; 26% with T2DM and 38% of obese had glucose in the range (5.6,6.9) mmol/L; and 67% with T2DM and 0% of obese had glucose above 6.9 mmol/L. Measurements for both T2DM and obese groups had significant positive correlation between HbA1c and glucose: *r* = 0.73 with *p* < 0.001 for the T2DM group and *r* = 0.37 with *p* = 0.020 for the obese group.

Further blood sample measurements are shown in Table 4. Cholesterol level was lower (*p* = 0.021), HDL level was lower (*p* = 0.047), and LDL level was lower (*p* < 0.001) for people in the T2DM group, while the triglyceride level was not significantly different (*p* = 0.221) between the two groups. Compared to reference ranges, 35% of people in the T2DM group and 51% of the obese group had cholesterol greater than 5.2 mmol/L, 33% of T2DM and 18% of obese had HDL less than 1.03 mmol/L, and 39% of people in the T2DM group and 70% of the obese group had LDL greater than 3.3 mmol/L. The measurements on people in both groups showed significant positive correlation between cholesterol and LDL, *r* > 0.87 with *p* < 0.001, and significant negative correlation between triglyceride and HDL, *r* < −0.45 with *p* < 0.005. The measurements on people in the T2DM group had a significant correlation between cholesterol and triglyceride, *r* = 0.33 with *p* = 0.042. The measurements on people in the obese group had significant correlations between cholesterol and HDL, *r* = 0.58 with *p* < 0.001, and between HDL and LDL, *r* = 0.45 with *p* = 0.005.

**Table 4:** Cholesterol, triglyceride, high-density lipoprotein (HDL), and low-density lipoprotein (LDL) measurements of participants with type 2 diabetes mellitus (T2DM) and the obese control group without diabetes

	Cholesterol (mmol/L)			Triglyceride (mmol/L)		
	T2DM	Obese	*p* value	T2DM	Obese	*p* value
Mean	4.97	5.56		2.00	1.70	
Std. Dev.	0.98	1.25		1.37	0.70	
Maximum	7.70	9.50	**0.021**	5.90	3.50	0.221
Median	5.00	5.40		1.50	1.40	
Minimum	2.80	3.20		0.40	0.70	
Number	43	39		40	39	
	HDL (mmol/L)			LDL (mmol/L)		
	T2DM	Obese	*p* value	T2DM	Obese	*p* value
Mean	1.24	1.38		3.03	3.69	
Std. Dev.	0.31	0.33		0.81	0.83	
Maximum	2.00	2.00	**0.047**	4.40	6.00	**< 0.001**
Median	1.30	1.40		3.10	3.50	
Minimum	0.60	0.80		1.10	2.00	
Number	42	39		41	37	

The *p* values were calculated using *t* tests

Bold values denote statistical significance at the *p* < 0.05 level

Table 5 displays clinical ophthalmic parameters. Between the two groups of participants, there were no significant differences in the Schirmer test or BUT, while the mean value of IOP was higher (*p* = 0.022) for people in the T2DM group. However, all three of these ophthalmic measurements were considered within normal limits.

**Table 5:** Ophthalmic clinical parameters of participants with type 2 diabetes mellitus (T2DM) and the obese control group without diabetes

	Intraocular pressure (mmHg)			Schirmer test (mm/5 min)			Tear film breakup time (sec)		
	T2DM	Obese	*p* value	T2DM	Obese	*p* value	T2DM	Obese	*p* value
Mean	16.9	15.3		10.9	8.8		6.8	8.6	
Std. Dev.	2.6	3.7		10.7	6.2		4.2	5.4	
Maximum	23.0	21.0	**0.022**	36.0	26.0	0.317	18.0	21.0	0.079
Median	17.0	15.0		6.5	8.0		6.5	8.0	
Minimum	12.0	8.0		1.0	1.0		1.0	1.0	
Number	44	39		38	33		44	38	

The *p* values were calculated using *t* tests

Bold values denote statistical significance at the *p* < 0.05 level

### Corneal nerve cell densities and corneal sub-basal plexus

The IVCCM data for the two groups of participants are summarized in Table 6. There were no significant differences in epithelial cell, stromal keratocyte, or endothelial cell densities (all *p* > 0.132) between T2DM and obese groups. In the T2DM group, the CN fiber length (*p* = 0.025), CN total branch density (*p* = 0.013), and CN fiber area (*p* = 0.009) values were significantly lower; there was a borderline significant difference in CN fiber width (*p* = 0.051); and no difference was seen in CN fiber density (*p* = 0.550), CN branch density (*p* = 0.088), and CN fractal dimension (*p* = 0.093). Among these corneal parameters, there were positive correlations among several pairs of CN fiber density, CN branch density, CN fiber length, CN total branch density, CN fiber area, and CN fractal dimension: 0.49 < *r* < 0.94 with *p* < 0.002 for measurements in the T2DM group and 0.59 < *r* < 0.93 with *p* < 0.019 for the obese group. The exception was CN fiber width that had negative correlation with CN fiber density for T2DM, *r* = −0.48 with *p* = 0.001, and with CN branch density for obese, *r* = −0.40 with *p* = 0.019. Pairwise, CN fiber length, CN total branch density, and CN fiber area are significantly positive correlated with each other, 0.69 < *r* < 0.76 with *p* < 0.001 for people with T2DM and 0.85 < *r* < 0.89 with *p* < 0.001 for obese. CN fiber width is not significantly correlated with CN fiber length, CN total branch density, or CN fiber area.

**Table 6:** In vivo corneal confocal microscopy (IVCCM) measurements of corneal confocal microscopy images and corneal nerve (CN) properties of participants with type 2 diabetes mellitus (T2DM) and the obese control group without diabetes

	Epithelial cell density (number/mm^2^)			Stromal keratocyte density (number/mm^2^)			Endothelial cell density (number/mm^2^)		
	T2DM	Obese	*p* value	T2DM	Obese	*p* value	T2DM	Obese	*p* value
Mean	6622	6726		387.6	441.9		3285	3544	
Std. Dev.	778	906		110.5	115.4		791	709	
Maximum	8083	8911	0.580	665.0	686.2	0.221	5551	5044	0.132
Median	6766	6674		359.3	441.3		3073	3645	
Minimum	5320	4623		202.0	216.0		1943	2171	
Number	43	38		43	39		42	37	
	CN fiber density (fibers/mm^2^)			CN branch density (fibers/mm^2^)			CN fiber length (mm/mm^2^)		
	T2DM	Obese	*p* value	T2DM	Obese	*p* value	T2DM	Obese	*p* value
Mean	12.05	12.93		12.43	16.49		9.61	11.27	
Std. Dev.	6.25	6.83		8.09	12.06		3.16	3.39	
Maximum	26.25	29.54	0.550	31.25	45.00	0.088	14.83	20.01	**0.025**
Median	11.87	11.46		9.82	13.75		10.11	10.14	
Minimum	1.56	2.50		2.68	1.04		1.42	6.31	
Number	42	37		40	35		44	37	
	CN total branch density (branch points/mm^2^)			CN fiber area (mm^2^/mm^2^)			CN fiber width (mm/mm^2^)		
	T2DM	Obese	*p* value	T2DM	Obese	*p* value	T2DM	Obese	*p* value
Mean	24.66	32.78		0.00491	0.00587		0.0229	0.0223	
Std. Dev.	12.37	16.04		0.00150	0.00168		0.0017	0.0010	
Maximum	51.78	69.88	**0.013**	0.00824	0.01017	**0.009**	0.0277	0.0245	0.051
Median	22.41	27.08		0.00482	0.00543		0.0228	0.0223	
Minimum	3.12	11.80		0.00130	0.00293		0.0193	0.0199	
Number	44	36		44	36		44	36	
	CN fractal dimension								
	T2DM	Obese	*p* value						
Mean	1.427	1.445							
Std. Dev.	0.049	0.042							
Maximum	1.502	1.518	0.093						
Median	1.439	1.441							
Minimum	1.307	1.349							
Number	43	37							

The *p* values were calculated using *t* tests

Bold values denote statistical significance at the *p* < 0.05 level

### Retinal nerve measurements

The OCT measurements are summarized in Table 7. Neither central retinal thickness nor RNFL values showed any differences between the T2DM and obese groups, except for a significant difference in RNFL nasal superior thickness (*p* = 0.010) and a borderline difference for RNFL nasal inferior thickness (*p* = 0.056). Foveal volume and choroidal thickness were not different between T2DM and obese groups. Among these OCT parameters, there were significant positive correlations (*p* < 0.05) between several of the pairs of the seven RNFL parameters and foveal volume in both T2DM and obese groups: 0.35 < *r* < 0.75 with *p* < 0.038. For the T2DM group, RNFL nasal superior was significantly correlated with RNFL global thickness, RNFL temporal superior, RNFL nasal, RNFL nasal inferior, and foveal volume: 0.35 < *r* < 0.63 with *p* < 0.019. For the obese group, RNFL nasal superior was significantly correlated only with RNFL global thickness: *r* = 0.48 with *p* = 0.003.

**Table 7:** Optical coherence tomography (OCT) measurements of retinal nerve fiber layer (RNFL) properties of participants with type 2 diabetes mellitus (T2DM) and the obese control group without diabetes

	Central retinal thickness (μm)			Central choroidal thickness (μm)			RNFL global thickness (μm)		
	T2DM	Obese	*p* value	T2DM	Obese	*p* value	T2DM	Obese	*p* value
Mean	275.0	272.05		249.7	227.4		100.0	104.4	
Std. Dev.	16.6	23.60		44.4	37.9		11.5	10.3	
Maximum	316.0	322.0	0.505	345.0	283.0	0.064	121.0	132.0	0.066
Median	278.0	273.0		242.5	233.5		102.5	103.0	
Minimum	240.0	225.0		165.0	135.0		75.0	76.0	
Number	43	39		30	22		44	39	
	RNFL temporal superior (μm)			RNFL temporal (μm)			RNFL temporal inferior (μm)		
	T2DM	Obese	*p* value	T2DM	Obese	*p* value	T2DM	Obese	*p* value
Mean	139.6	144.6		67.5	68.6		142.6	144.6	
Std. Dev.	19.2	22.5		10.6	9.7		20.6	16.7	
Maximum	176.0	190.0	0.271	96.0	86.0	0.634	200.0	183.0	0.636
Median	139.5	147.0		67.0	71.0		142.5	145.0	
Minimum	99.0	102.0		46.0	48.0		94.0	117.0	
Number	44	39		44	39		44	39	
	RNFL nasal superior (μm)			RNFL nasal (μm)			RNFL nasal inferior (μm)		
	T2DM	Obese	*p* value	T2DM	Obese	*p* value	T2DM	Obese	*p* value
Mean	112.1	123.4		77.9	79.5		113.6	123.2	
Std. Dev.	19.9	18.2		15.1	13.3		24.0	20.6	
Maximum	152.0	166.0	**0.010**	118.0	107.0	0.606	170.0	167.0	0.056
Median	112.0	123.0		76.0	79.5		111.0	121.0	
Minimum	79.0	82.0		52.0	44.0		73.0	68.0	
Number	44	37		44	38		44	39	
	Foveal volume 6 mm (mm^3^)								
	T2DM	Obese	*p* value						
Mean	8.61	8.55							
Std. Dev.	0.38	0.31							
Maximum	9.29	9.12	0.452						
Median	8.59	8.60							
Minimum	7.78	7.91							
Number	44	39							

The *p* values were calculated using *t* tests

Bold values denote statistical significance at the *p* < 0.05 level

## Discussion

Considering the two groups of participants in this study, obesity might eventually lead to the development of T2DM through insulin resistance [21]. Obese study participants who regularly took antidiabetics, such as metformin, might be present with impaired glucose tolerance (IGT); however, we did not examine the presence of IGT in our obese cohort, neither did we take into account its possible effects during data analysis. Another limitation of our study was the size of the study sample; however, according to our knowledge, a similar study was only done in animal model [23]. Larger future studies are warranted.

A measure of similarity between the two groups is that two-thirds of those with T2DM qualify as obese. However, as shown in Table 2, the difference between mean values of BMI of the T2DM and obese groups is statistically significant (*p* < 0.001). The HbA1c and glucose measurements shown in Table 3 and Figs. 1 and 2 identify strong differences between the groups (*p* < 0.001). Further differences between the groups appear in cholesterol and LDL measurements for the people who are obese without diabetes: 51% have cholesterol levels and 70% have LDL levels above normal values. Although the IOP mean values differ between the groups, the IOP, Schirmer test, and BUT are all within corresponding acceptable healthy range for both groups of participants.

There are no correlations at the significance level of *p* < 0.05 between the ages of the participants and any of the other measurements in this study, nor between BMI and any of the other measurements, corresponding to the participants in both groups on a participant level. There is a significant positive correlation between HbA1c and glucose values, but none of the other measurements in this study are correlated with either HbA1c or glucose at the significance level of *p* < 0.05. Similarly, cholesterol, triglyceride, HDL, and LDL measurements exhibit some mutual correlations among each other, but none of these four lipid measurement categories have measurements that are correlated with any of the other measurements in this study at the significance level of *p* < 0.05.

**Fig. 3:**
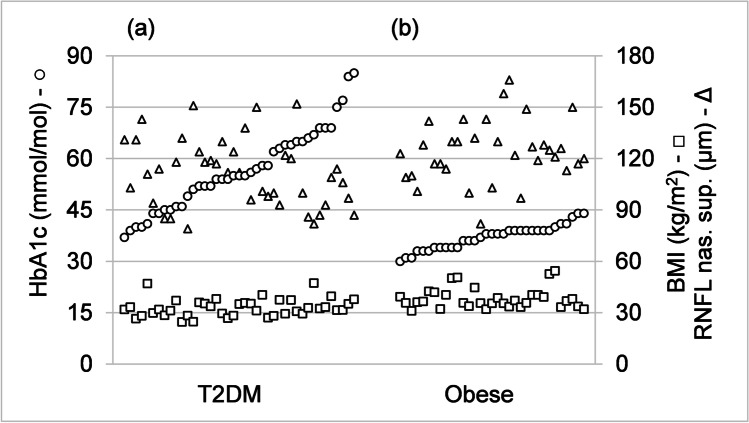
Measurements of HbA1c (mmol/mol), circles using left vertical scale, body mass index (BMI) (kg/m2), squares using right vertical scale, and retinal nerve fiber layer (RNFL) nasal superior (μm), triangles using right vertical scale, for **a** participants with type 2 diabetes mellitus (T2DM) (on the left) and **b** those who are obese without diabetes (on the right). The three measurements corresponding to a particular individual are drawn on a vertical line and the measurements are sorted within each group in ascending order of HbA1c along the horizontal axis. The resulting graph shows that HbA1c, BMI, and RNFL nasal superior are not mutually correlated (increased HbA1c is not accompanied by increases in BMI or RNFL nasal superior). T2DM, *N* = 41; obese, *N* = 33

For example, to illustrate lack of correlation, Fig. 3 provides HbA1c measurements plotted along with BMI and RNFL nasal superior using all measurements concurrently available for the three measurement categories separately from both T2DM and obese groups. Figure 3 is drawn with participants, people with T2DM on the left and obese on the right, as the horizontal axis and all measurements are sorted in ascending order with respect to HbA1c. Figure 3 shows that neither BMI nor RNFL nasal superior are correlated with the HbA1c measurements; for instance, participants with higher HbA1c do not correspondently have higher BMI or RNFL nasal superior values.

**Fig. 4:**
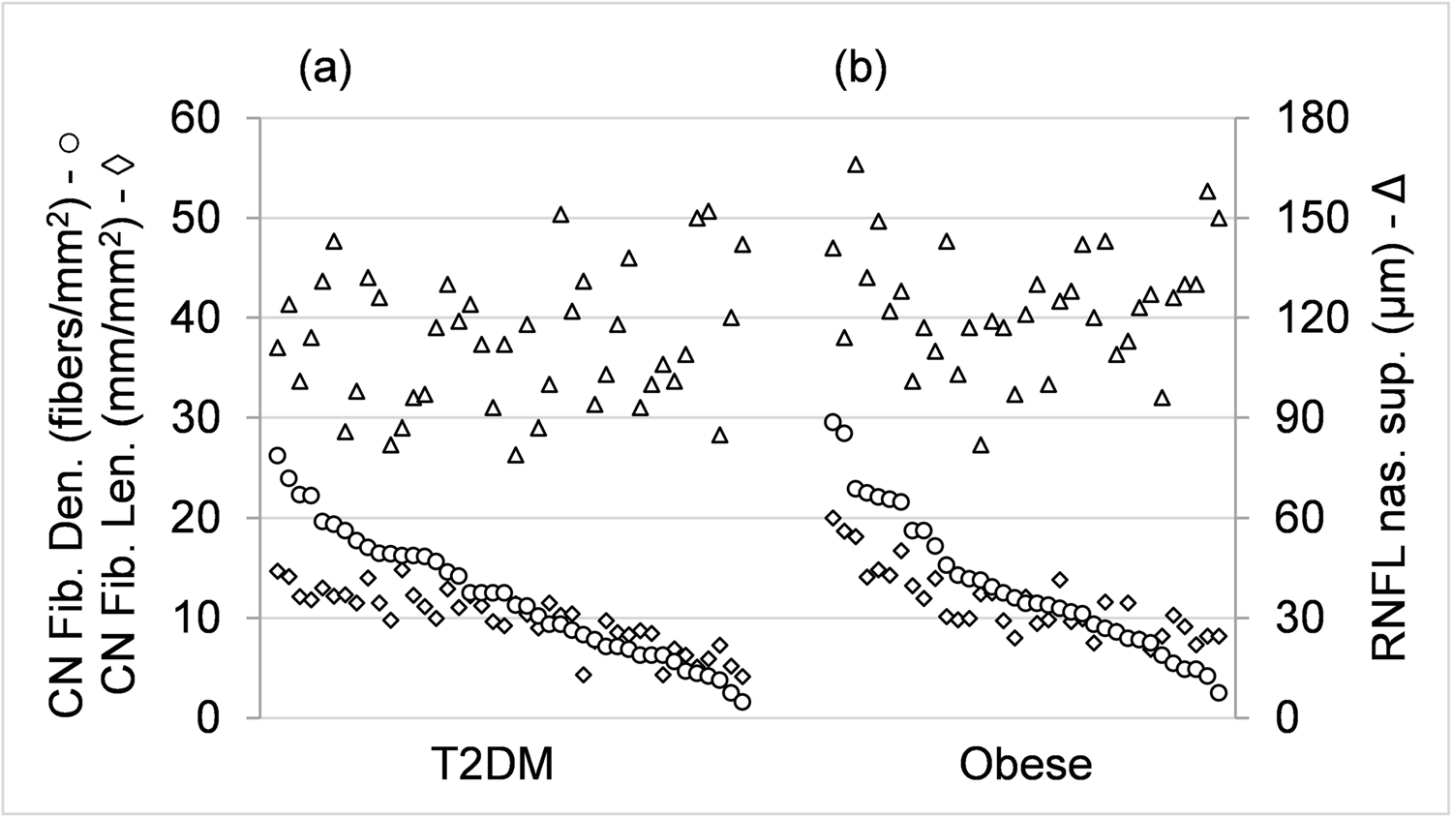
Measurements of corneal nerve (CN) fiber density (fibers/mm2), circles on left vertical scale, CN fiber length (mm/mm2), diamonds on left vertical scale, and retinal nerve fiber layer (RNFL) nasal superior (μm), triangles on right vertical scale for **a** participants with type 2 diabetes mellitus (T2DM) (on the left) and **b** those who are obese without diabetes on the right. The three measurements corresponding to a particular individual are drawn on a vertical line and the measurements are sorted within each group in descending order of CN fiber density along the horizontal axis. The results show that CN fiber density and CN fiber length are correlated but neither is correlated with RNFL nasal superior (decreases in CN fiber density are accompanied by a decrease in CN fiber length but not by a decrease in RNFL nasal superior). T2DM, *N* = 42; obese, *N* = 35

**Fig. 5:**
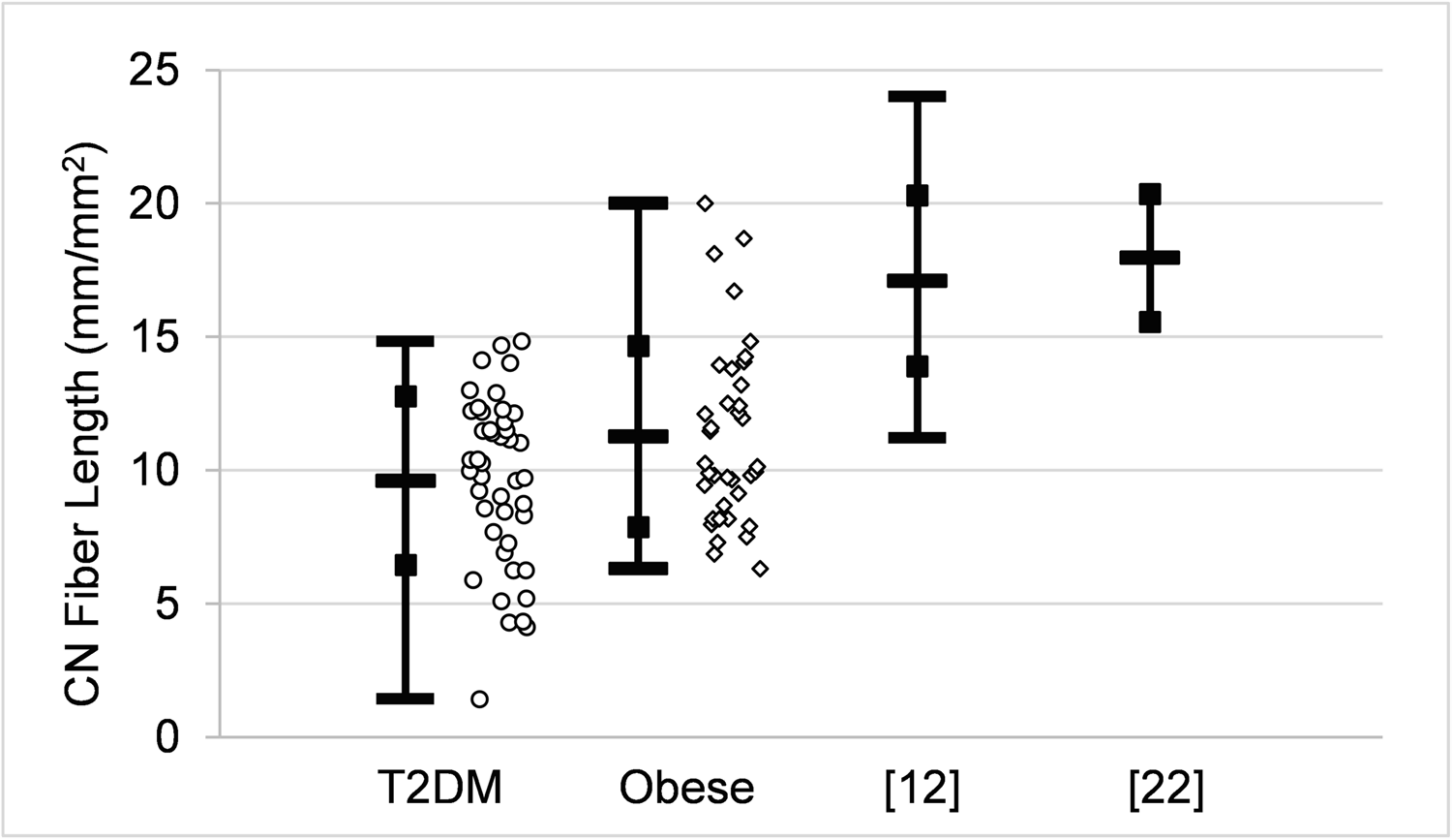
Comparison of measurements of corneal nerve (CN) fiber length (mm/mm^*2*^) for participants with type 2 diabetes mellitus (T2DM), circles, those who are obese without diabetes, diamonds, and two groups of people without diabetes or obesity from previous publications [12, 22]. Where the information is available, the open bars on vertical lines indicate mean value, open squares indicate ± standard deviation, and solid bars indicate minimum and maximum values. The raw data from the present work are shown scattered horizontally for clarity. T2DM, *N* = 44; obese, *N* = 37; [12], *N* = 48; [22], *N* = 12

For both T2DM and obese groups, there is significant correlation within the set of IVCCM measurements and separately within the set of OCT measurements. However, there is no significant correlation between IVCCM and OCT measurements at the significance level of *p* < 0.05. This is illustrated in Fig. 4, where CN fiber density, CN fiber length, and RNFL nasal superior measurements are plotted, similarly to Fig. 3, except sorted in descending order with respect to CN fiber density. Figure 4 shows a strong correlation between CN fiber density and CN fiber length (*r* = 0.821, *p* < 0.001 for T2DM and *r* = 0.846, *p* < 0.001 for obese) and that RNFL nasal superior measurements are not correlated with the CN fiber density or CN fiber length measurements; for instance, participants with lower CN fiber density have lower CN fiber length but they do not correspondently have lower RNFL nasal superior values. Normative IVCCM measurements of some corneal nerve fiber characteristics have been reported [12, 22]. Mean values for CN fiber density, CN branch density, and CN fiber length, shown in Table 6, are lower for both the T2DM group and the obese group for all three properties compared with previous works [12, 22]. This is illustrated in Fig. 5 showing the distribution of CN fiber length measurements: the difference between the mean values of the T2DM and obese groups is statistically significant, *p* = 0.025. Mean values of both the T2DM and obese groups are significantly different (lower) than the previously reported results [12, 22], *p* < 0.001. The previous mean values [12, 22] are not statistically different from one another, *p* = 0.312.

**Fig. 6:**
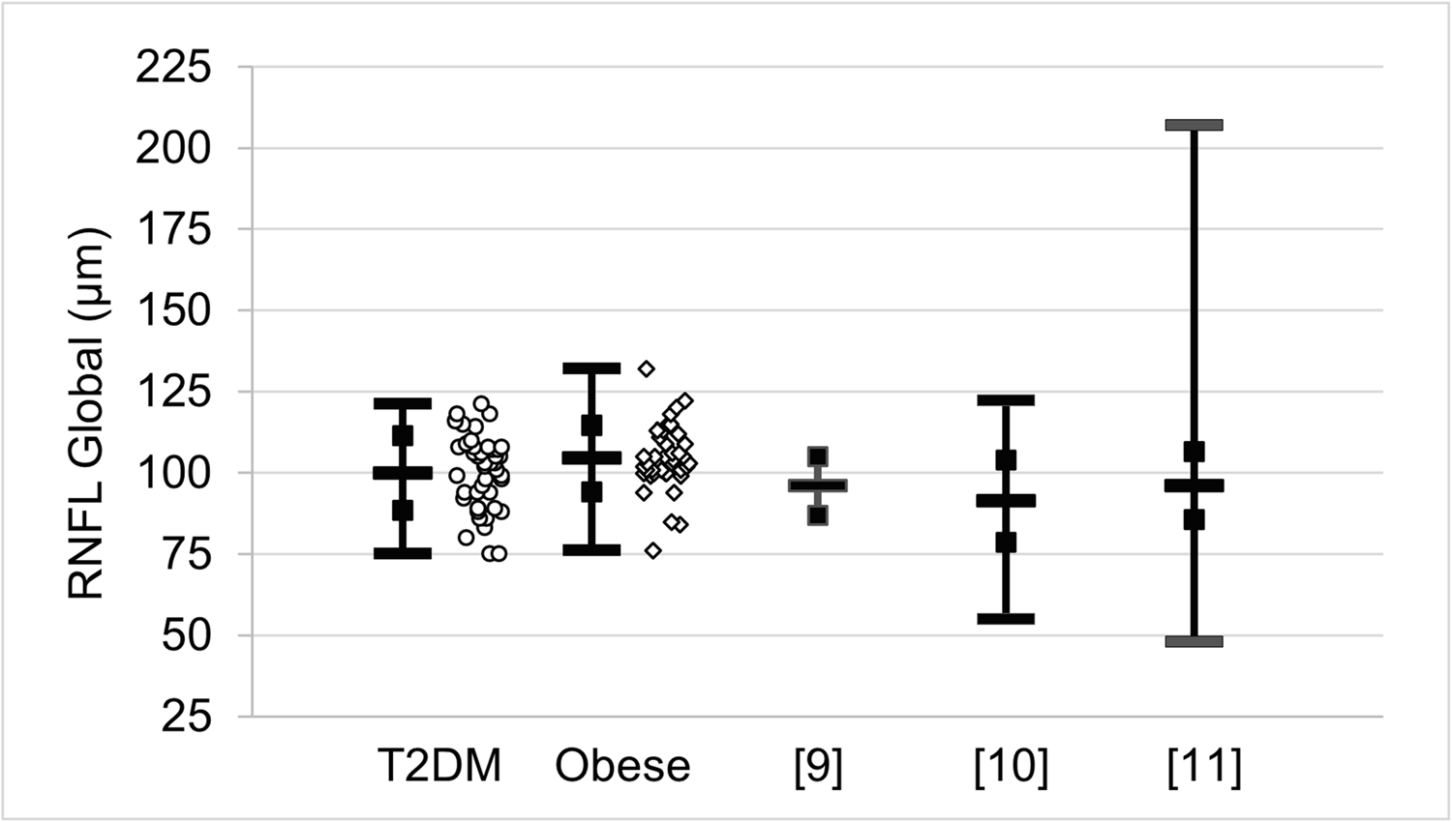
Comparison of measurements of retinal nerve fiber layer (RNFL) global (μm) for participants with type 2 diabetes mellitus (T2DM), circles, those who are obese without diabetes, diamonds, and three groups of people without diabetes or obesity from previous publications [9–11]. Where the information is available, the open bars on vertical lines indicate mean value, open squares indicate ± standard deviation, and solid bars indicate minimum and maximum values. The raw data from the present work are shown scattered horizontally for clarity. T2DM, *N* = 44; obese, *N* = 39; [9], *N* = 617; [10], *N* = 398; [11], *N* = 3224

OCT mean values for RNFL temporal for both the T2DM and obese groups, shown in Table 7, are lower than those reported in previous researches [9–11]. However, the ranges of previously reported RNFL mean values for global, nasal, superior, and inferior are all lower than the corresponding mean values in Table 7. This is illustrated in Fig. 6 showing the distribution of RNFL global measurements. The mean values for the T2DM and obese groups are not significantly different, *p* = 0.066, but both are significantly different (higher) than the previously reported results [9–11], *p* < 0.032.

The results in Table 7 show that the RNFL difference between the T2DM and obese participants was significant in the nasal superior quadrant and borderline in the nasal inferior. However, the connection between diabetes, complications of diabetes, and RNFL thickness values have not been fully elucidated to date. Some studies have shown multiple statistically significant relationships related to this issue [24, 25], but other studies have not [26], or just partially [27]. The roles of altered metabolic control and subclinical ischemia are presumed etiological factors in retinal neurodegeneration and axonal damages in T2DM [28]. OCT can be useful to detect these neurodegenerative changes, and as more and more information is gathered about the phenomenon, a better understanding of the process can make it easier to diagnose and assess progression and expected risk in complicated cases. In the present work using IVCCM and OCT, the analysis identifies four properties that show statistically significant differences between the mean values of measurements on people in the T2DM group and those in the obese control group: CN fiber length, CN total branch density, CN fiber area, and RNFL nasal superior. For each of the four properties, the measurement mean value for the T2DM group is less than that for the obese group, representing a possible degradation of nerve fiber status with diabetic state. However, CN fiber length, CN total branch density, and CN fiber area are pairwise significantly correlated with each other for both T2DM and obese groups, while RNFL nasal superior is not significantly correlated with any of these three CN parameters. Moreover, in general, the set of measurements of corneal nerve fiber are not correlated with the set of measurements of retinal nerve fiber with statistical significance at the level of *p* < 0.05. Although not a universal rule, the implication is that the progression from pre-diabetic (obese) to T2DM might tend to entail a loss or diminishment of certain corneal nerve fibers or retinal nerve fibers, but not necessarily a loss of both corneal and retinal nerve fibers at the same time.

This work identifies four nerve cell properties where the differences in mean values are statistically significant between T2DM and obese participants. Although these trends hold for comparisons of mean values between the groups, there is a high degree of overlap in the distribution of individual measurements between the groups, as illustrated in Figs. 5 and 6. Thus, the measured values of nerve cell properties do not by themselves distinguish between the two groups, in contrast with HbA1c and glucose (Figs. 1 and 2) that show a distinction between T2DM and obese. The practical significance is that each patient must be examined as a unique individual. Both IVCCM and OCT are useful for identifying nerve cell changes of interest and would need to be used over time to measure progression for a given individual.

Although glycemic control has been seen to improve some nerve parameters [22], loss of nerve fibers can lead to various ophthalmic dysfunctionalities, e.g., sensitivity impairment, and corneal erosion [29–31]. Therefore, it is important to monitor both the cornea and retina, with IVCCM and OCT, respectively, for remission or progressive damage to the eye in obese individuals and those with diabetes.

## Supplementary Information

ESM 1 (XLSX 36 kb)

## Data Availability

The [Supplementary_Table_S1] data used to support the findings of this study are included within the supplementary information file.
